# Safety and efficacy of oral fexinidazole in children with gambiense human African trypanosomiasis: a multicentre, single-arm, open-label, phase 2–3 trial

**DOI:** 10.1016/S2214-109X(22)00338-2

**Published:** 2022-09-27

**Authors:** Victor Kande Betu Kumesu, Wilfried Mutombo Kalonji, Clélia Bardonneau, Olaf Valverde Mordt, Digas Ngolo Tete, Séverine Blesson, François Simon, Sophie Delhomme, Sonja Bernhard, Pathou Nganzobo Ngima, Hélène Mahenzi Mbembo, Jean-Pierre Fina Lubaki, Steven Lumeya Vuvu, Willy Kuziena Mindele, Médard Ilunga Wa Kyhi, Guylain Mandula Mokenge, Lewis Kaninda Badibabi, Augustin Kasongo Bonama, Papy Kavunga Lukula, Crispin Lumbala, Bruno Scherrer, Nathalie Strub-Wourgaft, Antoine Tarral

**Affiliations:** a Ministry of Health, Kinshasa, Democratic Republic of the Congo; b National Human African trypanosomiasis Control Programme, Kinshasa, Democratic Republic of the Congo; c Department of Research and Development, Drugs for Neglected Diseases initiative, Kinshasa, Democratic Republic of the Congo; d Department of Research and Development, Drugs for Neglected Diseases initiative, Geneva, Switzerland; e Department of Medicine, Clinical Research Unit, Swiss Tropical and Public Health Institute, Basel, Switzerland; f Department of Medicine, University of Basel, Basel, Switzerland; g Bandundu Hospital, Kwilu, Democratic Republic of the Congo; h Vanga Hospital, Kwilu, Democratic Republic of the Congo; i Masi Manimba Hospital, Kwilu, Democratic Republic of the Congo; j Dipumba Hospital, Mbuji Mayi, Kasaï Oriental, Democratic Republic of the Congo; k Mushie Hospital, Maï Ndombe, Democratic Republic of the Congo; l Katanda Hospital, Kasaï Oriental, Democratic Republic of the Congo; m Isangi Hospital, Tshopo, Democratic Republic of the Congo; n Bagata Hospital, Kwilu, Democratic Republic of the Congo; o Bruno Scherrer Conseil, Saint Arnoult en Yvelines, France

## Abstract

**Background:**

Fexinidazole has been reported as an effective oral monotherapy against non-severe gambiense human African trypanosomiasis in a recent trial in adults. We aimed to assess the safety and efficacy of fexinidazole in children across all disease stages of gambiense human African trypanosomiasis.

**Methods:**

We did a multicentre, single-arm, open-label, phase 2–3 trial at eight district hospitals in the Democratic Republic of the Congo. We recruited children with a Karnofsky score of more than 50, those aged 6 years to younger than 15 years, weighing 20 kg or more, and with confirmed gambiense human African trypanosomiasis (any stage). Children weighing 20 kg or more and less than 35 kg received oral fexinidazole of 1200 mg (two × 600 mg tablets) once per day for 4 days (days 1–4) followed by 600 mg (one × 600 mg tablet) once per day for 6 days (days 5–10). Children weighing 35 kg or more received oral fexinidazole of 1800 mg (three × 600 mg tablets) once per day for 4 days (days 1–4), followed by 1200 mg (two × 600 mg tablets) once per day for 6 days (days 5–10). The primary endpoint was fexinidazole treatment success rate 12 months after end of treatment. A rate greater than 80% was deemed acceptable and a target value of 92% was aimed for. Safety was assessed through routine monitoring. This study is completed and registered with ClinicalTrials.gov, number NCT02184689.

**Findings:**

Between May 3, 2014, and Nov 22, 2016, we screened a total of 130 paediatric patients, of whom 125 (96%) received at least one dose of fexinidazole. All 125 patients (69 [55%] patients with stage 1, 19 [15%] with early stage 2, and 37 [30%] with late stage 2 gambiense human African trypanosomiasis) completed the 10-day treatment. Treatment success rate at 12 months was 97·6% (95% CI 93·1–99·5; 122 of 125 patients). The primary endpoint was met and the targeted value of 92% was exceeded. Treatment success at 12 months was elevated across all disease stages: 98·6% (95% CI 92·2–99·9; 68 of 69 patients) in stage 1, 94·7% (74·0–99·9; 18 of 19 patients) in early stage 2, and 97·3% (85·8–99·9; 36 of 37 patients) in late stage 2 gambiense human African trypanosomiasis. No new safety issues were observed beyond those found in adult trials. Overall, 116 (93%) of 125 patients reported 586 treatment-emergent adverse events, mainly mild or moderate. The most frequently reported treatment-emergent adverse events of interest during hospital admission were vomiting (86 [69%] of 125) and headache (41 [33%]). Seven (6%) of 125 patients had severe malaria, which was often accompanied by anaemia that was unrelated to fexinidazole. One patient died following dyspnoea and injury due to traumatic aggression 172 days after end of treatment, which was considered unrelated to fexinidazole or gambiense human African trypanosomiasis.

**Interpretation:**

Oral fexinidazole is a safe and effective first-line treatment option across all gambiense human African trypanosomiasis disease stages in paediatric patients.

**Funding:**

Through the Drugs for Neglected Diseases initiative: the Bill & Melinda Gates Foundation (USA), the Republic and Canton of Geneva (Switzerland), the Dutch Ministry of Foreign Affairs (Netherlands), the Norwegian Agency for Development Cooperation (Norway), the Federal Ministry of Education and Research through KfW (Germany), the Brian Mercer Charitable Trust (UK), and other private foundations and individuals from the human African trypanosomiasis campaign.

**Translation:**

For the French translation of the abstract see Supplementary Materials section.

## Introduction

Human African trypanosomiasis, or sleeping sickness, is a vector-borne parasitic disease transmitted by tsetse flies infected with one of two *Trypanosoma brucei* species. The most prevalent form of human African trypanosomiasis, which is due to *Trypanosoma brucei gambiense*, predominantly affects young adults involved in activities that facilitate human-to-vector contact. However, children are also exposed, particularly in riverine areas.^1^

**Research in context**

**Evidence before this study**

Fexinidazole, an oral gambiense human African trypanosomiasis treatment, was shown in a pivotal study to be acceptably safe and effective when compared with nifurtimox and eflornithine combination therapy for patients with late-stage gambiense human African trypanosomiasis in a randomised, non-inferiority trial. We did not do a systematic review before this study. Our study is part of a wider development programme started by Drugs for Neglected Diseases *initiative* (DND*i*) in 2005; therefore, most publications on fexinidazole clinical research for human African trypanosomiasis have been written within the DND*i* programme. A targeted review for this study considered paediatric human African trypanosomiasis, in different databases through a general Google search and also specifically in PubMed.

**Added value of this study**

The current multicentre study, designed in parallel to the pivotal trial and done in the same sites, aimed to demonstrate that fexinidazole safety and efficacy extends to a paediatric population, including those with CNS infection. The findings at the 12-month and 18-month follow-up visits support the use of oral fexinidazole to treat children, regardless of gambiense human African trypanosomiasis disease stage. The current study is the first trial to have focused specifically on a paediatric population. The primary endpoint was met, with a treatment success rate at 12 months in children who received fexinidazole of 97·6% (95% CI 93·1–99·5; 122 of 125 patients). The success rate exceeded both the 80% unacceptable rate and the targeted 92% success rate.

**Implications of all the available evidence**

For most patients, fexinidazole use avoids invasive lumbar puncture, associated with complications and anxiety, which is particularly pertinent for children. Because previously available gambiense human African trypanosomiasis treatments required substantial levels of health-care personnel and facilities, these treatments were limited to hospital settings for advanced infections. Fexinidazole fills an unmet need by being a safe, easily administered, oral treatment option that is effective across all stages of gambiense human African trypanosomiasis infection. Limitations include a small sample size (n=125) and the fact that the 10-day treatment course, the need for administration with food, and a high proportion of vomiting might lead to imperfect compliance. Further research, especially on home-based treatments, will help clarify whether this effectiveness is maintained in real-life conditions.

Soon after infection, the parasites proliferate in the haemolymphatic system and later invade the central nervous system. Unless treated, human African trypanosomiasis is usually fatal after a chronic progressive course.^2^ Few treatment options exist for human African trypanosomiasis. Early-stage disease has been treated with intramuscular pentamidine for *Trypanosoma brucei gambiense* and suramin for *Trypanosoma brucei rhodesiense*. Advanced neurological infection has been treated with melarsoprol, eflornithine, and, since 2009, nifurtimox combined with eflornithine.^3^

Although the prevalence of human African trypanosomiasis has decreased considerably over the past few years, with fewer than 1000 cases reported annually since 2018,^4^, ^5^ safe and easily administered therapies are needed that are effective across all stages of gambiense human African trypanosomiasis, without the requirement for disease staging via lumbar puncture for cerebrospinal fluid (CSF). This need has been partially met in the form of fexinidazole, an oral-only gambiense human African trypanosomiasis treatment repurposed from an antiparasitic agent initially developed in the 1970s.^6^ Fexinidazole was shown to be safe and effective for patients who were 15 years or older with late-stage gambiense human African trypanosomiasis in a pivotal multicentre, randomised, non-inferiority, phase 2–3 trial.^7^ In our study, we therefore aimed to assess whether fexinidazole's safety and efficacy profile also extends to a paediatric population with all stages of gambiense human African trypanosomiasis.

## Methods

### Study design and participants

We did a multicentre, single-arm, open-label, phase 2–3 trial at eight district hospitals in the Democratic Republic of the Congo, where the vast majority of reported cases (>80%) are diagnosed and treated.^8^ This trial was a plug-in to the pivotal study done simultaneously in the same centres.^7^

Our study included patients with a Karnofsky score of more than 50, those aged 6 years to younger than 15 years, weighing 20 kg or more, with confirmed gambiense human African trypanosomiasis, and able to swallow fexinidazole tablets with a solid meal. Stage 1 gambiense human African trypanosomiasis was defined as evidence of trypanosomes in the blood or lymph, no trypanosomes in the CSF, and white blood cell counts in the CSF of five cells or fewer per μL. Early stage 2 gambiense human African trypanosomiasis differs from stage 1 only by the CSF white blood cell count being more than five to 20 cells or fewer per μL. Late-stage 2 gambiense human African trypanosomiasis was defined as evidence of trypanosomes in the blood or lymph, and CSF white blood cell counts of more than 20 cells per μL or trypanosomes in the CSF.

Patients were excluded if they were severely malnourished according to the WHO 2007 growth reference data,^9^ pregnant or breastfeeding, had had treatment for human African trypanosomiasis in the 2 years before intake of fexinidazole, clinically significant laboratory test abnormalities, or electrocardiogram (ECG) abnormalities assessed by a cardiologist and Fridericia's corrected QT-interval (QTcF) of 450 ms or more (on two successive ECGs in a resting position, done 10–20 min apart). Patients were treated for soil transmitted helminthiasis, and were tested and, if necessary, treated for malaria.

The study protocol was approved by Comité d'éthique and Direction de la Pharmacie et des Medicaments, Ministry of Health of the Democratic Republic of the Congo, and the Comité de Protection des Personnes of Hôpital Necker (Paris, France), and is available online.^10^ The study was designed and done in accordance with the Declaration of Helsinki and the International Council for Harmonisation E6 Good Clinical Practice Guidelines. Informed consent forms were signed by a parent or legal representative. The child's assent to participate in the study was recorded in the presence of an impartial witness. An independent data safety monitoring board reviewed the study data regularly.

### Procedures

Fexinidazole was administered orally, in a dose regimen dependent on bodyweight. Children weighing 20 kg or more and less than 35 kg received 1200 mg (two × 600 mg tablets) once per day for 4 days (days 1–4), followed by 600 mg (one × 600 mg tablet) once per day for 6 days (days 5–10). Patients weighing 35 kg or more were given the adult dose—ie, 1800 mg (three × 600 mg tablets) administered orally, once per day for 4 days (days 1–4), followed by 1200 mg (two × 600 mg tablets) once per day for 6 days (days 5–10; appendix 2 p 1). Given the important interaction of fexinidazole with food, which increases absorption of the drug, treatment was administered within 30 min of the start of the main meal of the day, provided in the early morning and consisting of locally available food.

Temporary interruption of treatment was permitted for a maximum of 1 day with reintroduction at the investigator's discretion and an additional day of treatment added to compensate. Treatment was to be stopped in case of severe skin reaction; alanine aminotransferase (ALT) or aspartate aminotransferase (AST) of more than 8 × upper limit of normal (ULN); ALT or AST of more than 3 × ULN associated with total bilirubin of more than 2 × ULN; ALT or AST of more than 3 × ULN accompanied by fatigue, nausea, vomiting, right upper quadrant pain or tenderness, fever, rash, or eosinophilia (>5%); QTcF interval of 500 ms or more in two separate ECGs before intake of fexinidazole on days 2–5; or any condition that, in the opinion of the investigator, required treatment discontinuation for medical reasons.

Patients were observed for approximately 19 months. Study visits were scheduled on days 5 and 8 (during treatment), day 11 (end of treatment), days 13–18 (end of hospital admission, depending on the patient's clinical status), week 9 (for a subset of patients), months 3, 6, and 12 (primary efficacy timepoint), and month 18 (appendix 2 p 1). When needed, rescue treatment was nifurtimox and eflornithine combination therapy (oral nifurtimox [5 mg/kg three times per day for 10 days] and intravenous eflornithine [200 mg/kg twice per day for 7 days]). The full schedule of study procedures is available in appendix 2 (pp 2–3).

### Outcomes

The primary endpoint was fexinidazole treatment success rate 12 months after end of treatment. The minimum fexinidazole treatment success rate at 12 months was set at 80%. This cutoff value was chosen because fexinidazole was considered an acceptable treatment if the excess rate of success did not reach 13% in favour of nifurtimox and eflornithine combination therapy, based on a survey of gambiense human African trypanosomiasis clinicians.^7^ The success rate expected with the reference treatments at 12 months was 93%, estimated by combining the historical success rates of nifurtimox and eflornithine combination therapy in patients with stage 2 gambiense human African trypanosomiasis and pentamidine in patients with stage 1 gambiense human African trypanosomiasis.^11^, ^12^ The targeted rate for fexinidazole was set at 92%, which was higher than the expected rate in the pivotal study (89%),^7^ because the primary endpoint was at 12 months rather than 18 months, and patients with early-stage gambiense human African trypanosomiasis were included.

The primary treatment outcome was assessed at the test-of-cure visit, 12 months after end of treatment, using a more conservative approach to define success than the WHO criteria. The decision tree used to classify treatment success or failure depended on disease stage (appendix 2 pp 4–5). The outcome at 12 months was considered a success if the patient was alive, with no evidence of trypanosomes in any body fluid, and CSF white blood cell count of 20 cells or fewer per μL; the patient was alive, without lumbar puncture at 12 months, but with lumbar puncture at 18 months with favourable evaluation; or the patient was alive, without lumbar puncture at 12 months and 18 months, but with lumbar puncture at 6 months with favourable evaluation.

Secondary objectives included verifying whether fexinidazole treatment success rate varied depending on disease stage, whether fexinidazole treatment success rate depends on the white blood cell count in the CSF before treatment initiation, and the changes in success rate over time. Treatment outcomes were assessed at 24 h, 6 months, 12 months (primary timepoint), and 18 months after end of therapy.

Adverse events were reported by the patient or the investigator. Adverse events of interest were headache, vomiting, neutropenia, depression, and anxiety, following results obtained in patients with Chagas disease.^13^ Human African trypanosomiasis signs and symptoms, ECGs, physical and neurological or psychiatric examination, vital signs, standard haematology, and blood chemistry were also recorded (appendix 2 p 6). Adverse events were also reported by their severity, using the Common Terminology Criteria for Adverse Events grading scale (version 4.03). Serious adverse events were reported separately from severe adverse events according to the different definition for clinical trials. A serious adverse event was any event that resulted in death, was life-threatening, required in-patient hospitalisation or prolongation of existing hospitalisation, resulted in persistent or substantial disability or incapacity, was a congenital anomaly or birth defect, or was another important medical event that might have jeopardised the patient or might have required intervention to prevent one of the other outcomes listed in the definition above.

### Statistical analysis

Determination of the sample size was based on the primary analysis of the primary efficacy endpoint. Two hypotheses were tested simultaneously. The first hypothesis estimated the true success rate being 80% or less (ie, the unacceptable rate) and the second hypothesis estimated the true success rate being 92% or more (ie, the targeted or expected rate for fexinidazole). With a sample size of 125 patients, the probability of rejecting the first hypothesis was 97·5% if the true success rate is 92% with a one-sided type I error of 0·025. The probability of rejecting the second hypothesis was also 97·5% if the true success rate was 80% with a one-sided type I error of 0·025.

In the intention-to-treat population (ie, all patients who received at least one dose of fexinidazole), the success rate at 12 months was computed with a two-sided 95% CI using the Clopper-Pearson method. The lower limit of the confidence interval was compared with the unacceptable rate of 80%. Descriptive safety analyses were done on the intention-to-treat population. Treatment success at 12 months according to disease stage was calculated with the Fisher exact test, and the relationship between the success rate at 12 months and white blood cell count in CSF with logistic regression in the intention-to-treat population. The time course of treatment response rate was presented as a Kaplan-Meier curve based on patients who did not have definitive treatment failure. The comparison of success rates at the follow-up visits was done using a Cochran *Q* test. The effect of time adjusted for site was also tested with a mixed model for repeated measures.

All summaries and statistical analyses were generated using SAS (version 9.2 or higher). This study is completed and registered with ClinicalTrials.gov, NCT02184689.

### Role of the funding source

The funders of the study had no role in the study design, data collection, data interpretation, or writing of the report.

## Results

Between May 3, 2014, and Nov 22, 2016, we screened a total of 130 patients for the study; of these patients, five (4%) were not included (four patients with stage 1 human African trypanosomiasis and one patient with late-stage 2 human African trypanosomiasis; figure). Reasons for non-inclusion were meeting exclusion criteria (four patients: abnormal ECG [n=1], QTcF ≥450 ms [n=2], and pregnancy [n=1]) and withdrawal of consent (n=1). Of the 130 paediatric patients screened, 125 (96%) who received at least one dose of fexinidazole were included in the intention-to-treat population. All these patients completed the 10-day treatment. Of these 125 patients, 69 (55%) had stage 1 gambiense human African trypanosomiasis, 19 (15%) had early stage 2 gambiense human African trypanosomiasis, and 37 (30%) had late stage 2 gambiense human African trypanosomiasis. Mean age was 10·81 years (SD 2∙31). 103 (82%) of 125 patients weighed 20 kg to less than 35 kg, and 22 (18%) weighed 35 kg or more. BMI values (<15·0 kg/m^2^ in 94 [75%] of 125 patients) and low albumin and blood urea nitrogen concentrations were consistent with poor nutritional state. Respective mean CSF white blood cell count values in patients with stage 1 gambiense human African trypanosomiasis was 2∙87 cells per μL, those with early stage 2 gambiense human African trypanosomiasis was 10∙84 cells per μL, and late-stage 2 gambiense human African trypanosomiasis was 223∙81 cells per μL (table 1).

**Figure:**
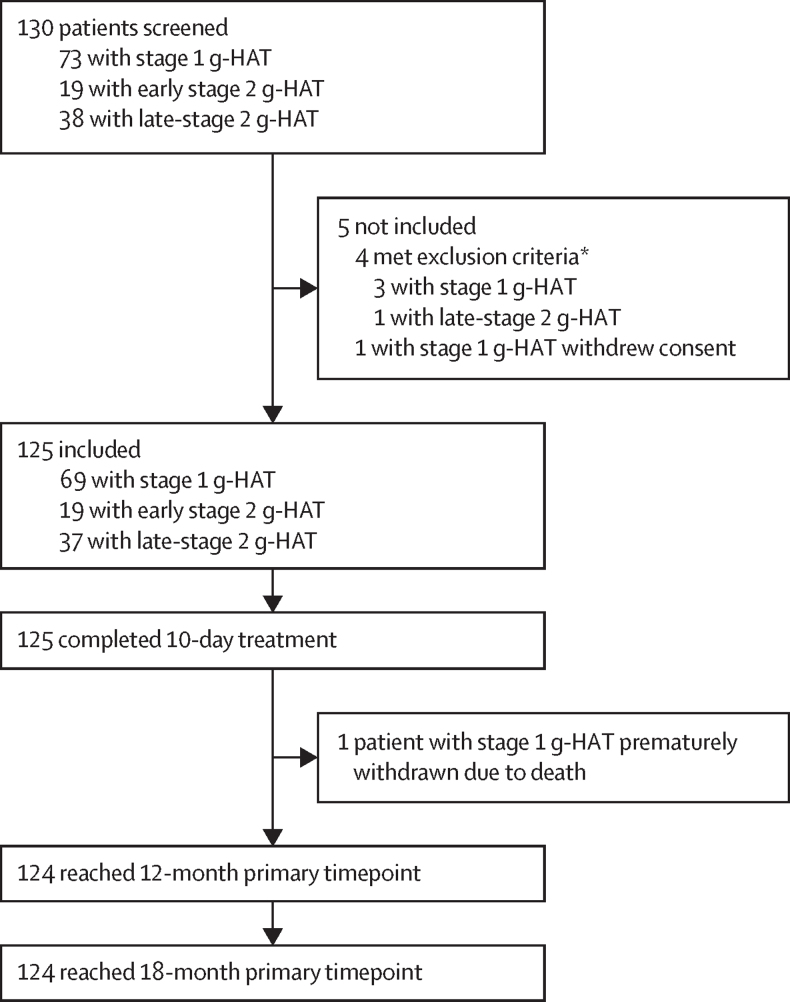
Study flowchart of complete 18-month data g-HAT=gambiense human African trypanosomiasis. QTcF=Fridericia's corrected QT-interval. *Three patients had an abnormal ECG and one patient was pregnant.

**Table 1:** Baseline characteristics in the intention-to-treat population

		**Stage 1 g-HAT (n=69)**	**Early stage 2 g-HAT (n=19)**	**Late-stage 2 g-HAT (n=37)**	**Total (n=125)**
**Demographics**					
Female		36 (52%)	7 (37%)	15 (41%)	58 (46%)
Male		33 (48%)	12 (63%)	22 (59%)	67 (54%)
Age (years)		10·96 (2·33)	10·79 (2·23)	10·54 (2·34)	10·81 (2·31)
Weight (kg)		29·23 (7·71)	27·89 (7·88)	26·80 (6·78)	28·30 (7·49)
BMI (kg/m^2^)		16·09 (1·48)	15·94 (1·55)	16·06 (1·38)	16·06 (1·45)
**Parasitological findings**					
Tests performed					
	Blood examination	69 (100%)	19 (100%)	37 (100%)	125 (100%)
	Lymph examination	29 (42%)	9 (47%)	18 (49%)	56 (45%)
	Positive CATT	66 (96%)	18 (95%)	32 (86%)	116 (93%)
	Positive HAT rapid diagnostic test	2/57 (4%)	2/17 (12%)	2 (5%)	6/111 (5%)
	Positive lymph node sampling	20 (29%)	8 (42%)	14 (38%)	42 (34%)
	Positive CTC	23 (33%)	7 (37%)	3 (8%)	36 (29%)
	Positive mAECT	22 (32%)	2 (11%)	2/36 (6%)	26/124 (21%)
	Positive mAECT-BC	2/56 (4%)	3/17 (18%)	8/36 (22%)	13/109 (12%)
	CSF examination	69 (100%)	19 (100%)	37 (100%)	125 (100%)
Method used to test for CSF trypanosomes					
	Direct	29/67 (43%)	3 (16%)	14 (38%)	46/123 (37%)
	Single centrifugation	36/67 (54%)	16 (84%)	23 (62%)	75/123 (61%)
	Direct and single centrifugation	2 (3%)	0	0	2 (2%)
	Positive trypanosomes or CSF	0	0	25 (68%)	25 (20%)
	CSF white blood cell count (cells per μL)	2·87 (1·22)	10·84 (4·62)	223·81 (188·74)	69·48 (143·00)
**Vital signs and general health**					
Systolic blood pressure (mm Hg)		99·04 (9·74)	98·95 (12·86)	100·70 (11·50)	99·52 (10·73)
Diastolic blood pressure (mm Hg)		64·35 (9·47)	67·37 (9·33)	66·89 (7·64)	65·56 (8·98)
Temperature (°C)		36·48 (0·42)	36·53 (0·52)	36·63 (0·67)	36·53 (0·52)
Heart rate (beats per min)		84·20 (11·55)	82·74 (19·01)	91·22 (16·12)	86·06 (14·58)
Respiratory rate per min		21·33 (3·14)	21·68 (4·36)	23·46 (5·10)	22·02 (4·08)
Altered general health		5 (7%)	1 (5%)	17 (46%)	23 (18%)

Data are n (%), n/N (%), or mean (SD). CATT=card agglutination test for trypanosomiasis. CTC=capillary tube centrifugation. HAT=human African trypanosomiasis. g-HAT=gambiense HAT. mAECT=mini-anion exchange centrifugation technique. mAECT-BC=mini-anion exchange centrifugation technique on buffy coat.

Consistent with severity of infection at baseline, cervical adenomegaly was more prevalent in patients with late stage 2 gambiense human African trypanosomiasis (24 [65%] of 37) than in those with early stage 2 (seven [37%] of 19) or stage 1 (25 [36%] of 69) gambiense human African trypanosomiasis. The most frequently reported signs and symptoms of the disease at baseline were headache (69 [55%] of 125), fever (56 [45%] of 124), weight loss (48 [38%]), drowsiness (42 [34%]), asthenia (39 [31%]), and pruritus (27 [22%]). Most patients had a normal neuropsychiatric examination (75 [60%] of 125) at inclusion. Abnormalities reported in more than 5% of patients at baseline included disrupted rapid alternating movements (22 [18%] of 124), volitional tremor (13 [10%] of 125), positive Romberg test (nine [7%] of 124), behavioural disorder (eight [6%] of 125), lethargy (eight [6%] of 125), involuntary movements (eight [6%] of 125), presence of palmomental reflex (eight [6%] of 125), and difficulties walking (seven [6%] of 125).

Major protocol deviations were reported in two (2%) of 125 patients; one of these patients had a history of human African trypanosomiasis treatment in the past 2 years, and one patient had treatment deviation (ie, 18 tablets of fexinidazole were administered instead of 14 tablets). Six (5%) of 125 patients received a higher dose of fexinidazole than planned at least once (including one major deviation previously mentioned), and one (1%) patient received a lower dose than planned. 124 (99%) of 125 patients reached the 12-month and 18-month timepoints except for one (1%) patient, who died during the study (details about this death is discussed further below; figure).

The treatment success rate at 12 months was 97·6% (95% CI 93·1–99·5; 122 of 125 patients). The primary endpoint was met and the targeted value of 92% was exceeded; the lower limit of the 95% CI was greater than the 80% unacceptable rate. Three treatment failures at 12 months were reported, including the one aforementioned patient who died of unrelated causes. Two patients had a CSF white blood cell count of more than 20 cells per μL at 12 months (one patient with early stage 2 infection at inclusion and one patient with late stage 2 infection). Treatment success rate at 12 months was acceptable across all stages of gambiense human African trypanosomiasis: 98·6% (95% CI 92·2–99·9; 68 of 69 patients) in stage 1, 94·7% (74·0–99·9; 18 of 19 patients) in early stage 2, and 97·3% (85·8–99·9; 36 of 37 patients) in late-stage 2. No significant relationship was observed between success rate at 12 months and white blood cell count in the CSF at screening. The analysis of the timing of failure did not show any trend over time. Treatment success was sustained at 18 months, and was 98·4% (95% CI 93·4–99·8; 123 of 125 patients), with two failures: the CSF white blood cell count of one of the patients with treatment failure at 12 months, who was clinically stable and thus was not prescribed a rescue treatment, fell below 20 cells per μL in the final 18-month visit and became a success.

The overall incidence of signs and symptoms of gambiense human African trypanosomiasis at the end of hospital admission was lower than at inclusion (appendix 2 p 6): headache (three [2%] of 125 *vs* 69 [55%] of 125), fever (three [2%] *vs* 56 [45%]), weight loss (14 [11%] *vs* 48 [38%]), drowsiness (three [2%] *vs* 42 [34%]), asthenia (six [5%] *vs* 39 [31%]), and pruritus (five [4%] *vs* 27 [22%]). Despite some fluctuations after end of hospital admission, the reduction was maintained during the 18-month follow-up period. Of note, headache (11 [9%] of 124) and fever (eight [6%]) were still present in patients at the end of the 18-month follow-up.

116 (93%) of 125 patients reported 586 treatment-emergent adverse events during the study. 113 (90%) of 125 patients had treatment-emergent adverse events during the 10-day treatment period, which were mild or moderate in 115 (92%) of 125 patients (table 2; appendix 2 pp 7–9). Treatment-emergent adverse events considered possibly related to treatment were reported in 103 (82%) of 125 patients. Severe treatment-emergent adverse events were reported in 25 (20%) of 125 patients (table 3). The most frequently reported treatment-emergent adverse events (ie, >10%) were vomiting (86 [69%] of 125), nausea (47 [38%]), headache (41 [33%]), asthenia (39 [31%]), decreased appetite (24 [19%]), tremor (24 [19%]), salivary hypersecretion (18 [14%]), abdominal pain (15 [12%]), and anaemia (14 [11%]). Seven (6%) of 125 patients had severe malaria, which was often accompanied by anaemia that was unrelated to fexinidazole. One treatment-emergent adverse event of vomiting in a patient with late stage 2 gambiense human African trypanosomiasis led to temporary treatment discontinuation.

**Table 2:** Incidence of treatment-emergent adverse events and serious adverse events

		**Stage 1 g-HAT (n=69)**		**Early stage 2 g-HAT (n=19)**		**Late-stage 2 g-HAT (n=37)**		**Total (n=125)**	
		n (%)	Number of events	n (%)	Number of events	n (%)	Number of events	n (%)	Number of events
At least one adverse event		61 (88%)	317	18 (95%)	75	37 (100%)	195	116 (93%)	587
At least one treatment-emergent adverse event		61 (88%)	316	18 (95%)	75	37 (100%)	195	116 (93%)	586
	At least one treatment-emergent adverse event during the treatment period	60 (87%)	294	18 (95%)	69	35 (95%)	151	113 (90%)	514
	At least one treatment-emergent adverse event after the treatment period	17 (25%)	22	4 (21%)	6	18 (49%)	44	39 (31%)	72
	At least one treatment-emergent adverse event leading to treatment discontinuation	0	0	0	0	1 (3%)	1	1 (1%)	1
	At least one treatment-emergent adverse event leading to permanent treatment discontinuation	0	0	0	0	0	0	0	0
	At least one mild or moderate treatment-emergent adverse event	60 (87%)	306	18 (95%)	68	37 (100%)	182	115 (92%)	556
	At least one severe treatment-emergent adverse event	9 (13%)	10	5 (26%)	7	11 (30%)	13	25 (20%)	30
	At least one treatment-emergent adverse event possibly related to treatment	54 (78%)	207	17 (90%)	45	32 (86%)	101	103 (82%)	353
At least one serious adverse event		5 (7%)	6	2 (11%)	3	4 (11%)	8	11 (9%)	17

Data are from the complete 18-month timepoint of the intention-to-treat population (n=125). Causality was assessed by both the Investigator as planned in the protocol: a possibly-related adverse event was any event that was not considered as unrelated to the study treatment or for which no plausible alternative explanation existed. The funder of the study also assessed adverse event causality using this definition. The intensity of an adverse event was graded as mild (grade 1), moderate (grade 2), or severe (grade 3 was severe, grade 4 was life-threatening, and grade 5 was death). g-HAT=gambiense human African trypanosomiasis.

**Table 3:** All treatment-emergent adverse events from grade 3 to 5

		**Grade 3**		**Grade 4**		**Grade 5**		**Total**	
		n (%)	Number of events	n (%)	Number of events	n (%)	Number of events	n (%)	Number of events
Any adverse events		22 (18%)	25	2 (2%)	3	1 (1%)	2*	25 (20%)	30
Blood and lymphatic system disorders		7 (6%)	7	..	..	..	..	7 (6%)	7
	Anaemia	6 (5%)	6	..	..	..	..	6 (5%)	6
	Neutropenia	1 (1%)	1	..	..	..	..	1 (1%)	1
Infections and infestations		7 (6%)	7	..	..	..	..	7 (6%)	7
	Malaria	6 (5%)	6	..	..	..	..	6 (5%)	6
	Cerebral malaria	1 (1%)	1	..	..	..	..	1 (1%)	1
Investigations		4 (3%)	4	1 (1%)	2	..	..	5 (4%)	6
	Blood potassium increased	3 (2%)	3	..	..	..	..	3 (2%)	3
	Blood potassium decreased	1 (1%)	1	1 (1%)	1	..	..	2 (2%)	2
	Blood calcium decreased	..	..	1 (1%)	1	..	..	1 (1%)	1
Gastrointestinal disorders		4 (3%)	4	..	..	..	..	4 (3%)	4
	Vomiting	3 (2%)	3	..	..	..	..	3 (2%)	3
	Gastritis	1 (1%)	1	..	..	..	..	1 (1%)	1
Psychiatric disorders		2 (2%)	2	..	..	..	..	2 (2%)	2
	Psychotic disorder	2 (2%)	2	..	..	..	..	2 (2%)	2
General disorders and administration site conditions		1 (1%)	1	..	..	..	..	1 (1%)	1
	Pyrexia	1 (1%)	1	..	..	..	..	1 (1%)	1
Injury, poisoning, and procedural complications		..	..	..	..	1 (1%)	1	1 (1%)	1
	Injury	..	..	..	..	1 (1%)	1	1 (1%)	1
Metabolism and nutrition disorders		..	..	1 (1%)	1	..	..	1 (1%)	1
	Hyperkalaemia	..	..	1 (1%)	1	..	..	1 (1%)	1
Respiratory, thoracic, and mediastinal disorders		..	..	..	..	1 (1%)	1	1 (1%)	1
	Dyspnoea	..	..	..	..	1 (1%)	1	1 (1%)	1

Data are from the complete 18-month timepoint of the intention-to-treat population (n=125). Common Terminology Criteria for Adverse Events grade 3 corresponds to severe, grade 4 corresponds to life-threatening, and grade 5 corresponds to death.

* One patient died following two events (dyspnoea and injury due to traumatic aggression), which was considered unrelated to fexinidazole or gambiense human African trypanosomiasis.

A total of 17 treatment-emergent serious adverse events were reported in 11 (9%) of 125 patients. All serious adverse events started after end of treatment, and only one serious adverse event of asymptomatic blood potassium increased in one patient with stage 1 gambiense human African trypanosomiasis was considered possibly related to the treatment (table 3). This serious adverse event resolved spontaneously without sequelae. One (1%) of 125 patients with stage 1 gambiense human African trypanosomiasis died 172 days after end of treatment due to dyspnoea and injury caused by traumatic aggression; this death was considered unrelated to treatment or gambiense human African trypanosomiasis.

Of the five types of adverse events of interest, the most frequently observed during the hospitalisation period were vomiting and headache, as previously mentioned. Neutropenia was reported in four (3%) of 125 patients during hospital admission, of which one event was severe. Anxiety was less frequent, reported in two (2%) of 125 patients. No events of depression were reported.

An increase in the incidence of treatment-emergent adverse events from stage 1 gambiense human African trypanosomiasis through to late-stage 2 gambiense human African trypanosomiasis was noted for psychiatric disorders. Psychiatric adverse events were reported in one (1%) of 69 patients with stage 1 gambiense human African trypanosomiasis, two (11%) of 19 patients with early stage 2 gambiense human African trypanosomiasis, and 16 (43%) of 37 patients with late-stage 2 gambiense human African trypanosomiasis (table 4). In particular, 12 (10%) of 125 patients had insomnia and 24 (19%) had tremors, with a clear influence of gambiense human African trypanosomiasis stage (none in 69 patients with stage 1, one [5%] of 19 patients with early stage 2, and 11 [30%] of 37 patients with late stage 2 for insomnia; and 11 [16%] of 69 patients with stage 1, four [21%] of 19 patients with early stage 2, and nine [24%] of 37 patients with late stage 2 for tremor). Psychiatric and neurological examinations did not reveal any new safety signals, and a general improvement was observed during treatment, regardless of gambiense human African trypanosomiasis stage. In addition, an increase in infections and in skin and subcutaneous tissue disorders were reported in patients with late-stage 2 gambiense human African trypanosomiasis (table 4).

**Table 4:** Summary of treatment-emergent adverse events reported in at least 2% of patients

	**Stage 1 g-HAT (n=69)**		**Early stage 2 g-HAT (n=19)**		**Late-stage 2 g-HAT (n=37)**		**Total (n=125)**	
	n (%)	Number of events	n (%)	Number of events	n (%)	Number of events	n (%)	Number of events
Any adverse event	61 (88%)	316	18 (95%)	75	37 (100%)	195	116 (93%)	586
Gastrointestinal disorders	55 (80%)	165	15 (79%)	34	28 (76%)	58	98 (78%)	257
General disorders and administration site conditions	28 (41%)	34	7 (37%)	10	16 (43%)	21	51 (41%)	65
Investigations	12 (17%)	15	1 (5%)	3	7 (19%)	7	20 (16%)	25
Psychiatric disorders	1 (1%)	1	2 (11%)	2	16 (43%)	24	19 (15%)	27
Metabolism and nutrition disorders	10 (14%)	12	1 (5%)	1	13 (35%)	14	24 (19%)	27
Blood and lymphatic system disorders	10 (14%)	10	4 (21%)	5	6 (16%)	7	20 (16%)	22
Musculoskeletal and connective tissue disorders	8 (12%)	8	0	0	5 (14%)	7	13 (10%)	15
Infections and infestations	3 (4%)	3	2 (11%)	2	8 (22%)	11	13 (10%)	16
Eye disorders	7 (10%)	7	1 (5%)	1	2 (5%)	2	10 (8%)	10
Respiratory, thoracic, and mediastinal disorders	5 (7%)	7	0	0	1 (3%)	1	6 (5%)	8
Skin and subcutaneous tissue disorders	2 (3%)	3	0	0	5 (14%)	5	7 (6%)	8
Cardiac disorders	2 (3%)	3	1 (5%)	1	1 (3%)	1	4 (3%)	5

Data are from the complete 18-month timepoint of the intention-to-treat population (n=125). g-HAT=gambiense human African trypanosomiasis.

Gastrointestinal adverse events—particularly vomiting and nausea—were the most frequently reported events overall in 98 (78%) of 125 patients (table 4). Over the whole treatment period, 25 (20%) of 125 patients vomited mostly once within 30 min of receiving fexinidazole, causing treatment readministration. Two (2%) of 125 patients had treatment readministered after vomiting between 30 min and 2 h of receiving fexinidazole. In total, 76 (61%) of 125 patients vomited more than 2 h after fexinidazole administration.

Assessment of laboratory values, vital signs, single ECG, physical examination, and urinalysis did not raise any safety concerns. Although the trial was ongoing, a visit was added at 9 weeks, which included complete haematology and biochemistry examinations; 90 (74%) of 125 patients were tested for neutropenia at this visit, with a mean absolute neutrophil count of 2334 cells per μL (SD 966) of blood. Only one moderate adverse event of neutropenia was found at that visit (absolute neutrophil count of 546 cells per μL), which was considered possibly related to fexinidazole treatment and had resolved by the end of the study.

## Discussion

This study is the first clinical trial on human African trypanosomiasis (sleeping sickness) to have focused specifically on a paediatric population (aged ≥6 years weighing at least 20 kg). Previous trials have included children (IMPAMEL II^14^ and NECT Field^15^) but as part of the total study population and without a specific analysis. In our study, the primary endpoint was met, with a treatment success rate at 12 months in patients who received fexinidazole of 97·6% (95% CI 93·1–99·5; 122 of 125 patients). This success rate exceeded both the 80% unacceptable rate and the targeted 92% success rate. The consistency of the findings at 12 months and at the end of the 18-month follow-up period supports the use of fexinidazole as an oral drug to treat children, regardless of gambiense human African trypanosomiasis disease stage.

Fexinidazole treatment in our paediatric population did not raise any new safety signals compared with the pivotal trial.^7^ Although the incidence of vomiting was very common and proportionally higher in children than in adults at the beginning of treatment during the peak of drug concentration, vomiting tended to decrease over time during the maintenance phase (treatment readministration was required at some point in 27 [22%] of 125 patients). Potential concerns exist with respect to adherence, such as the requirement for fexinidazole to be administered during or after a solid meal in order to achieve efficacious concentrations, the relatively long dosing schedule for an oral treatment of 10 days, the change in the number of tablets midway through the dosing schedule, and nausea and vomiting being frequent side-effects.^16^, ^17^, ^18^, ^19^ A study including a subcohort on home-based treatment to help assess these difficulties is ongoing (NCT03025789).

The differences observed between stages are known and expected (eg, higher incidence of neuropsychiatric disorders, particularly insomnia, in patients with late stage 2 gambiense human African trypanosomiasis). Comparisons between disease stages need to be interpreted with caution because of the small patient numbers in the advanced-stage infection. Fexinidazole safety findings were consistent with the profile established in adults.^7^ Asymptomatic reversible neutropenia and liver injury that were observed at higher and longer dose regimens in adult patients treated for Chagas disease^13^ were not reported in children with the treatment regimen used in the present study. Only five (4%) of 125 patients, four during treatment and one at the 9-week visit, had a single event of neutropenia (one severe), without liver injury.

This study is limited by its single-arm, hence open-label, study design. A comparative double-blind study would have been difficult because of the different reference treatments for each stage and modes of administration. Moreover, comparison with pentamidine (stage 1) and nifurtimox and eflornithine combination therapy (stage 2) at sufficient power would require a large number of patients, which were not available in this age group. The duration of follow-up for the assessment of the primary endpoint was set at 12 months (6 months shorter than in the pivotal study),^7^ to expedite the granting of a scientific opinion by the European Medicines Agency, although this follow-up duration reduces comparability with other studies that precisely follow WHO's methodological framework for human African trypanosomiasis clinical trials. This shortened follow-up duration was considered acceptable because of the low relapse rate expected between 12 months and 18 months (approximately 1·2% based on historical data with melarsoprol, eflornithine,^20^ and nifurtimox and eflornithine combination therapy^12^), and because additional follow-up was done until 18 months after end of treatment. However, because of eventual late relapses, WHO still recommends a follow-up of 24 months after treatment in the latest guidelines.^21^

In our study, success rate at 18 months was close to that observed at 12 months, suggesting that the results at 12 months could be used to assess efficacy. Assessment of treatment success was highly conservative. Death, regardless of the cause, as well as possible loss to follow-up, were considered as failure. The risk of observing false successes was limited by the multiple approaches to assess post-treatment outcome (clinical, parasitological, and biological), and by technical supervision. If WHO's success criteria are applied, the efficacy increases to 99·2% (95% CI 95·62–99·98; 123 of 124 patients), as the study reported a death unrelated to the disease or the study drug as failure.^22^, ^23^ Another patient classified as having treatment failure could be defined as a probable relapse since parasites were not detected after treatment and during follow-up, and a raised white blood cell count (34 cells per μL in the CSF) was only observed at the 18-month visit compared with 201 white blood cells per μL in the CSF at screening.

The causes of adverse events are difficult to assess in patients with human African trypanosomiasis,^15^ because the events are often related and confused with the symptoms of the disease itself,^24^ or with concomitant diseases that are expected in sick and often malnourished patients. The appearance of symptoms absent at baseline was rare, except for vomiting, indicating that the signs and symptoms reported during the study were a consequence of the disease itself rather than a treatment side-effect. However, pharmacovigilance activities should be facilitated once fexinidazole is regularly used by the endemic countries' health systems.

The assessments in the present study align with those used in the pivotal study, although some scales were not specifically designed for children (eg, Karnofsky index) and some neurological and psychiatric dimensions might be more challenging to assess in young children (eg, anxiety or depression). However, the homogeneity of the methodology was necessary for pooling data from the pivotal study^7^ and to support generalisability or extrapolation of findings to children.

In conclusion, orally administered fexinidazole showed high efficacy across all stages of gambiense human African trypanosomiasis infection in children aged 6 years and older and weighing more than 20 kg. The benefit-to-risk ratio of fexinidazole for treating children with gambiense human African trypanosomiasis, regardless of disease stage, is positive. Current interventions for diagnosing, staging, and treating gambiense human African trypanosomiasis require resources, trained personnel, equipment, and hospital infrastructure. These potentially costly procedures are therefore difficult to implement in remote areas or in those that might be mired in conflict, which could prevent the goal of eliminating gambiense human African trypanosomiasis by 2030.^25^, ^26^ Simplified oral treatments such as fexinidazole or single-dose oral treatments such as acoziborole (currently in clinical trials)^27^ that can cure both disease stages of gambiense human African trypanosomiasis and circumvent the need for systematic disease staging with lumbar puncture (a procedure associated with complications and anxiety, particularly in children^28^) would benefit both patients and health-care professionals.^29^

## Data sharing

The data underlying the results of this study are available upon request. Interested researchers might contact the DND*i* commissioner of this study for data access requests via email at CTdata@dndi.org. Researchers might also request data by completing the form available online, in which interested researchers will confirm that they will share data and results with DND*i* and will publish any results as open access.

## Declaration of interests

BS reports personal fees from Drugs for Neglected Diseases *initiative* (DND*i*) during the conduct of the study and personal fees from DND*i* outside the submitted work. WMK, CB, OVM, SBl, FS, SD, NS-W, and AT report employment at DND*i*. All other authors declare no competing interests.

## Supplementary Materials

French translation of the abstract
