# Supplementary material for: Safety and efficacy of oral fexinidazole in children with gambiense human African trypanosomiasis: a multicentre, single-arm, open-label, phase 2–3 trial
Source: Lancet Glob Health. 2022 Sep 27;10(11):e1665–74. doi: 10.1016/S2214-109X(22)00338-2 (PMC9554014; doi:10.1016/S2214-109X(22)00338-2)
Supplement: French translation of the abstract [file mmc1.pdf]

THE LANCET
Global Health

Supplementary appendix 1

This translation in French was submitted by the authors and we reproduce it as supplied. It has not been peer reviewed. *The Lancet*'s editorial processes have only been applied to the original in English, which should serve as reference for this manuscript.

Cette traduction en français a été proposée par les auteurs et nous l'avons reproduite telle quelle. Elle n'a pas été examinée par des pairs. Les processus éditoriaux du *Lancet* n'ont été appliqués qu'à l'original en anglais et c'est cette version qui doit servir de référence pour ce manuscrit.

Supplement to: Kande Betu Kumesu V, Mutombo Kalonji W, Bardonneau C, et al. Safety and efficacy of oral fexinidazole in children with gambiense human African trypanosomiasis: a multicentre, single-arm, open-label, phase 2–3 trial. *Lancet Glob Health* 2022; published online Sept 27. https://doi.org/10.1016/S2214-109X(22)00338-2.

Généralités Une étude récente ayant montré l'efficacité du fexinidazole en monothérapie orale contre la trypanosomiase humaine Africaine à *T.b. gambiense* (THA-g) non grave chez l'adulte, nous avons cherché à évaluer la sécurité et l'efficacité du fexinidazole chez l'enfant, à tous les stades de la THA-g.

Méthodes Nous avons réalisé une étude de phase 2-3 multicentrique, ouverte et à un seul bras, dans huit hôpitaux de district de la République démocratique du Congo. Nous avons recruté des enfants âgés de 6 à < 15 ans, avec un poids corporel ≥20 kg et un score de Karnofsky > 50, atteints de THA-g confirmée (quel que soit le stade). Les enfants dont le poids corporel était compris entre 20 et <35 kg ont reçu 1200 mg (deux comprimés de 600 mg) de fexinidazole oral une fois par jour pendant 4 jours (jours 1 à 4), suivi de 600 mg (un comprimé de 600 mg) une fois par jour pendant 6 jours (jours 5 à 10). Les enfants pesant 35 kg ou plus ont reçu 1800 mg (trois comprimés de 600 mg) de fexinidazole oral une fois par jour pendant 4 jours (jours 1 à 4), suivi de 1200 mg (deux comprimés de 600 mg) une fois par jour pendant 6 jours (jours 5 à 10). Le critère d'évaluation principal était le taux de réussite du traitement par le fexinidazole 12 mois après la fin du traitement. Un taux supérieur à 80 % a été jugé acceptable, et la valeur cible a été fixée à 92 %. La sécurité du traitement a été évaluée par une surveillance de routine. Cette étude est terminée et enregistrée auprès de ClinicalTrials.gov sous le numéro NCT02184689.

Résultats Entre le 3 mai 2014 et le 22 novembre 2016, nous avons examiné un total de 130 patients pédiatriques, dont 125 (96%) ont reçu au moins une dose de fexinidazole. Tous les 125 patients, parmi lesquels 69 [55%] étaient au stade 1 de la THA-g, 19 [15%] au stade 2 précoce, et 37 [30%] au stade 2 tardif, ont terminé le traitement de 10 jours. Le taux de réussite du traitement à 12 mois était égal à 97,6% (soit 122 patients sur 125 ; IC à 95% : 93,1-99,5). Le critère d'évaluation principal a été atteint et la valeur cible de 92 % a été dépassée. Le taux de réussite du traitement à 12 mois était élevé à tous les stades de la maladie : 98,6% pour le stade 1 (soit 68 patients sur 69 ; IC à 95% : 92,2-99,9), 94,7% pour le stade 2 précoce (soit 18 patients sur 19 ; IC à 95% : 74,0-99,9) ; et 97-3% pour le stade 2 avancé (soit 36 patients sur 37 ; IC à 95% 85,8-99,9).

Aucun nouveau problème de sécurité n'a été observé, autres que ceux constatés dans les études menées chez l'adulte. Dans l'ensemble, 116 patients sur 125 (93 %) ont signalé 586 événements indésirables survenus au cours du traitement, principalement légers ou modérés. Les événements indésirables survenus au cours du traitement, et présentant un intérêt particulier, les plus

fréquemment signalés pendant l'hospitalisation étaient des vomissements chez 86 patients sur 125 (69 %), et des céphalées chez 41 patients sur 125 (33 %). Un paludisme grave a affecté 7 patients sur 125 (6 %), souvent accompagné d'une anémie, sans rapport avec le fexinidazole. Un patient est décédé 172 jours après la fin du traitement des suites de dyspnée et traumatisme provoqué par une agression, ce qui a été considéré comme non lié au fexinidazole, ou à la trypanosomiase humaine Africaine à *T.b. gambiense*.

Interprétation Le fexinidazole oral est une option thérapeutique de première intention, sûre et efficace contre tous les stades de la trypanosomiase africaine à *T.b. gambiense* chez les patients pédiatriques.

Financement Par le biais de Drugs for Neglected Diseases *initiative :* La Fondation Bill & Melinda Gates (Etats Unis), La République et Canton de Genève (Suisse), Le Ministère des Affaires Etrangères (Pays Bas), L'agence Norvégienne pour la Coopération au Développement (Norvège), Le Ministère Fédéral pour l'Education et la Recherche via KfW (Allemagne); le Brian Mercer Charitable Trust (RU), et d'autres fondations privées et donations individuelles de la campagne pour la trypanosomiase humaine Africaine.
