# Supplementary material for: Safety and efficacy of oral fexinidazole in children with gambiense human African trypanosomiasis: a multicentre, single-arm, open-label, phase 2–3 trial
Source: Lancet Glob Health. 2022 Sep 27;10(11):e1665–74. doi: 10.1016/S2214-109X(22)00338-2 (PMC9554014; doi:10.1016/S2214-109X(22)00338-2)
Supplement: Supplementary appendix 2 [file mmc2.pdf]

THE LANCET
Global Health

Supplementary appendix 2

This appendix formed part of the original submission and has been peer reviewed. We post it as supplied by the authors.

Supplement to: Kande Betu Kumesu V, Mutombo Kalonji W, Bardonneau C, et al. Safety and efficacy of oral fexinidazole in children with gambiense human African trypanosomiasis: a multicentre, single-arm, open-label, phase 2–3 trial. *Lancet Glob Health* 2022; published online Sept 27. https://doi.org/10.1016/S2214-109X(22)00338-2.

Supplementary Materials

Figure S1. Study design

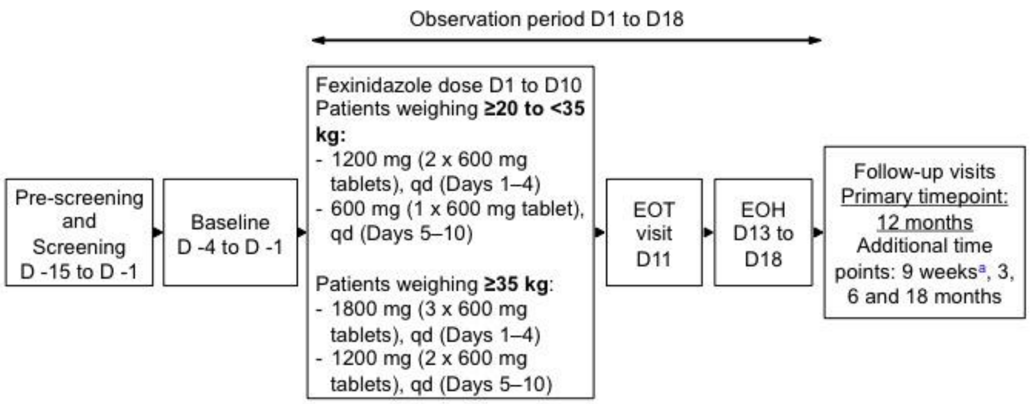

Patients were hospitalised from their arrival at the investigational site until Day 18 after treatment start (unless their clinical status was satisfactory, and they were permitted to leave from Day 13). Patients were followed for 18 months. [a]The Week 9 visit was performed in a subset of patients. D: day; EOH, end of hospitalisation; EOT, end of treatment.

Table S2. Schedule of study procedures

**Procedures**	**Pre-screening and screening**	**Baseline**	**Treatment Period**										**EOT visit until EOH visit**			**Follow-up Period**	
Time point	D-15 to D-1	D-4 to D-1	D1	D2	D3	D4	D5	D6	D7	D8	D9	D10	D11	D12	D13-D18	Week 9	3, 6, 12, 18 months
Blood and/or lymph sampling for detection of trypanosomes	X												X				X
CSF sampling (lumbar puncture) for detection of trypanosomes and WBC count	X												X				X
Informed consent (before any additional medicines or study-specific procedures)	X	Check															
Pre-treatment of helminthiasis (+3-day recovery period)	X																
Rapid diagnostic test and/or thick blood smear for malaria	X																
Pre-treatment of malaria if necessary (+3-day recovery period)	X																
Karnofsky score	X	Check					X			X			X		X	X	X
Safety ECG (single)	X	Check		X	X	X							X				
Urine pregnancy test		X													X		X
Inclusion/exclusion criteria	X	X															
Demographic data	X																
Medical history	X																X
Signs and symptoms of HAT		X													X		X
Vital signs	X	X					X			X			X		X	X	X
Physical and neurological examinations		X					X			X			X		X	X	X
Haematology/chemistry		X					X			X			X			X	X
Urinalysis		X											X				
ECGs for QT/QTcF assessment (triplicate)		X				X						X					
Fexinidazole administration			X	X	X	X	X	X	X	X	X	X					
Adverse events		X	X	X	X	X	X	X	X	X	X	X	X	X	X		
Serious adverse events		X	X	X	X	X	X	X	X	X	X	X	X	X	X	X	X
Prior/concomitant medications	X	X	X	X	X	X	X	X	X	X	X	X	X	X	X		
PK assessment: dry blood spot												X	X	X			
PK assessment: dry CSF spot													X				

Note: "Check" means that the test was to be performed if not done at pre-screening/screening, and it was to be redone in case of abnormal findings.

- The EOT visit assessments could be performed on Day 11 or Day 12.
- Patients were hospitalised from the time of their arrival at the investigational site until the EOH visit on Day 13 to Day 18. They were permitted to leave the hospital from Day 13 onwards, if their clinical status was considered satisfactory.
- Additional unscheduled visits could occur. If a relapse was suspected on the basis of physical examination or CSF WBC count, the patient returned for a visit within 1 to 3 months, at the discretion of the Investigator. The patient was also asked to return to the investigational site if he/she felt unwell, even if there was no apparent relationship with treatment and/or HAT. Assessments to be performed at unscheduled visits were: physical and neurological examination; HAT signs and symptoms; investigation of concomitant conditions; blood and lymph testing for trypanosomes (if indicated); CSF sampling to test for trypanosomes and WBC count (if symptoms suggesting disease progression were present); and additional safety assessments, at the discretion of the Investigator.
- A Week 9 visit was held (relative to Day 1) between Day 64 and Day 70. This visit was performed in a sub-sample of patients (protocol amendment 2, release date 25 August 2015).
- Follow-up visits were held (relative to EOT) at 3 months (±1 week), 6 months (±2 weeks), 12 months (±4 weeks), and 18 months (±4 weeks).
- Lumbar puncture at EOT was performed on Day 11 (24 hours after the last dose). Dry CSF spot at EOT was performed on the first 30 patients. If no failure was diagnosed at EOT on the first 30 patients, and exposure reached target levels, the following patients were not to be sampled. If at least one failure was diagnosed at EOT on the first 30 patients, sampling was to occur in all patients.
- Performed at 6, 12 and 18 months. CSF sampling was performed at 3 months if the patient presented with clinical signs or symptoms of HAT.
- Additional safety assessments such as ECGs and clinical laboratory tests could be performed, at the discretion of the Investigator.
- ECG was transmitted electronically for central reading. Inclusion of the patient in the study was conditional on receiving the report from the cardiologist, confirming the patient was eligible.
- Performed in girls aged 12 years and older. The first test (Baseline) was performed on Day -1.
- Was to be performed only at the 3-month visit.
- Only the new events since last visit were documented.
- Triplicate ECG readings, taken 2 minutes apart, were recorded at: baseline; Day 4 (4 hours and 23 hours post-dose); and Day 10 (between 2 and 3 hours post-dose). The ECG measurements on Day 4, at 23 hours post-dose (ie, 1 hour before study treatment administered on Day 5) were also used as a safety assessment; if QTcF was >500 ms, a second ECG was performed after a 10- to 20-minute rest. If the value was confirmed, the patient was withdrawn from the study, and no further doses of fexinidazole were administered.
- Moreover, any adverse event occurring after the period of adverse event reporting, and considered by the Investigator to be possibly related to study drug, was also to be reported.
- Including treatment history.
- Blood sampling was performed on: Day 10 (3 hours and 7 hours, 15 minutes post-dose), on Day 11 (24 hours after the last dose) and on Day 12 (48 hours after the last dose).
- At EOT, dried spot CSF sampling was to be performed on the first 30 children. The CSF sample that was collected for the efficacy assessment was also used for PK assessment by dry spot testing at: Day 11: 24 hours post dose (relative to last dose on Day 10).

CSF, cerebrospinal fluid; D, day; ECG, electrocardiogram; EOH, end of hospitalisation; EOT, end of treatment; HAT, Human African Trypanosomiasis; QT, QT interval on ECG (interval of time between electrical depolarisation and repolarisation of the right and left cardiac ventricles); QTcF, QT interval corrected by heart rate, according to the Fridericia formula; PK, pharmacokinetic; WBC, white blood cell.

Figure S3. Algorithm of classification to categorise treatment success for the primary efficacy endpoint (12 months)

- Stage 1 and early stage 2 g-HAT

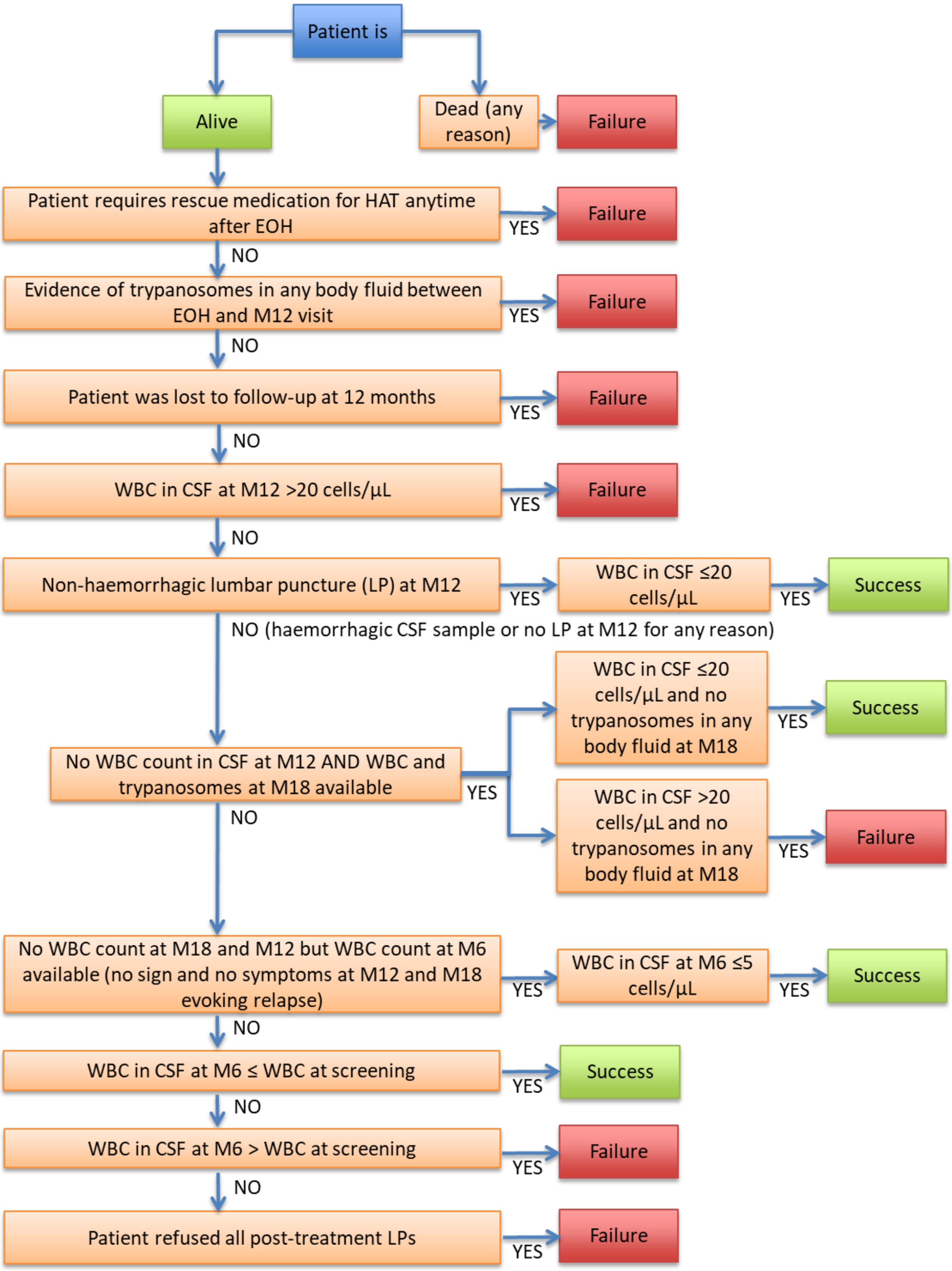

- Late stage 2 g-HAT

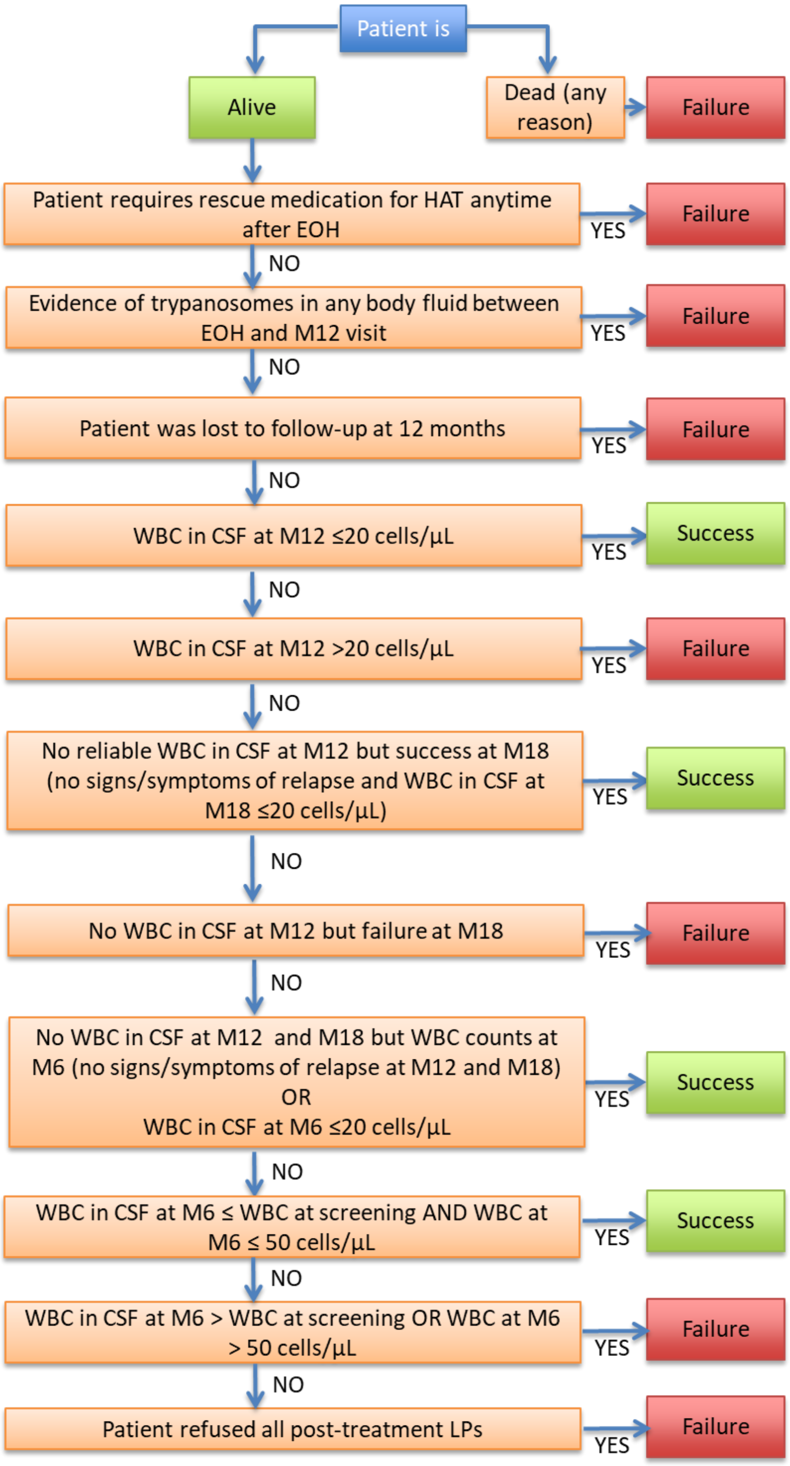

Figure S4. HAT clinical signs and symptoms over time: radar chart at inclusion and EOH (ITT population)

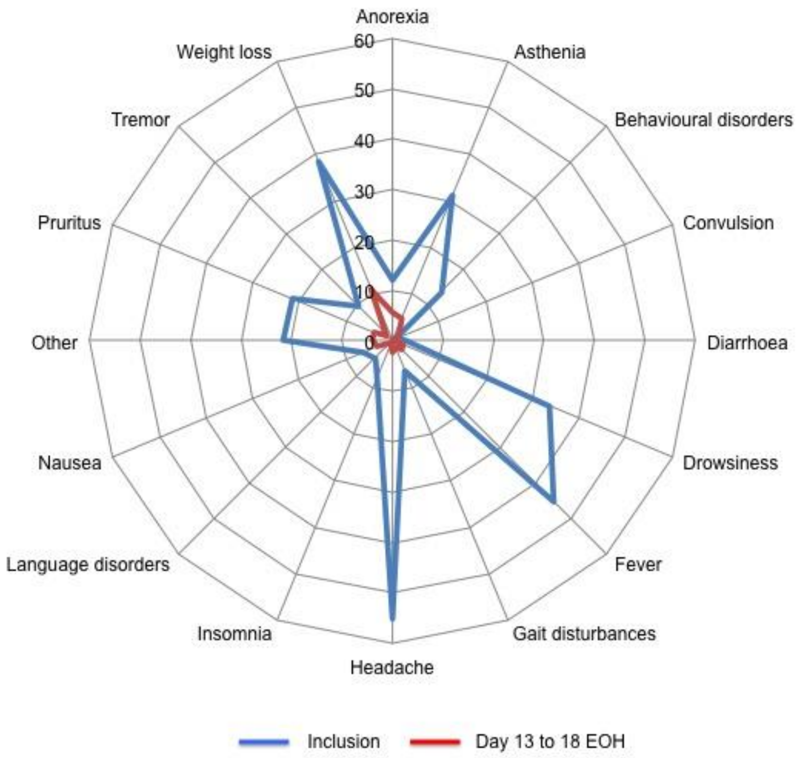

D, Day; EOH, end of hospitalisation; HAT, Human African Trypanosomiasis; ITT, intention-to-treat.

Table S5. All adverse events by system organ class and preferred term according to intensity (complete 18-month data, ITT population)

	Grade 1	Grade 2	Grade 3	Grade 4	Grade 5	Total
**Any adverse events**	**109 (87·2%) [444]**	**59 (47·2%) [113]**	**22 (17·6%) [25]**	**2 (1·6%) [3]**	**1 (0·8%) [2]**	**116 (92·8%) [587]**
**Gastrointestinal disorders**	**91 (72·8%) [221]**	**22 (17·6%) [32]**	**4 (3·2%) [4]**			**98 (78·4%) [257]**
Vomiting	74 (59·2%) [106]	19 (15·2%) [25]	3 (2·4%) [3]			86 (68·8%) [134]
Nausea	45 (36·0%) [57]	2 (1·6%) [2]				47 (37·6%) [59]
Salivary hypersecretion	17 (13·6%) [19]	1 (0·8%) [1]				18 (14·4%) [20]
Abdominal pain	13 (10·4%) [14]	3 (2·4%) [3]				15 (12·0%) [17]
Dyspepsia	7 (5·6%) [8]					7 (5·6%) [8]
Dysphagia	6 (4·8%) [7]					6 (4·8%) [7]
Gastritis	5 (4·0%) [5]		1 (0·8%) [1]			6 (4·8%) [6]
Abdominal pain upper	2 (1·6%) [2]					2 (1·6%) [2]
Diarrhoea	2 (1·6%) [2]					2 (1·6%) [2]
Dry mouth	1 (0·8%) [1]					1 (0·8%) [1]
Submaxillary gland enlargement		1 (0·8%) [1]				1 (0·8%) [1]
**Nervous system disorders**	**51 (40·8%) [76]**	**16 (12·8%) [20]**				**61 (48·8%) [96]**
Headache	31 (24·8%) [38]	10 (8·0%) [12]				41 (32·8%) [50]
Tremor	19 (15·2%) [21]	5 (4·0%) [5]				24 (19·2%) [26]
Dizziness	11 (8·8%) [12]					11 (8·8%) [12]
Movement disorder	2 (1·6%) [2]					2 (1·6%) [2]
Cerebellar syndrome	1 (0·8%) [1]					1 (0·8%) [1]
Convulsion		1 (0·8%) [1]				1 (0·8%) [1]
Extrapyramidal disorder	1 (0·8%) [1]					1 (0·8%) [1]
Grand mal convulsion		1 (0·8%) [1]				1 (0·8%) [1]
Psychomotor hyperactivity	1 (0·8%) [1]					1 (0·8%) [1]
Somnolence		1 (0·8%) [1]				1 (0·8%) [1]
**General disorders and administration site conditions**	**45 (36·0%) [54]**	**8 (6·4%) [10]**	**1 (0·8%) [1]**			**51 (40·8%) [65]**
Asthenia	36 (28·8%) [41]	3 (2·4%) [3]				39 (31·2%) [44]
Pyrexia	6 (4·8%) [7]	4 (3·2%) [5]	1 (0·8%) [1]			11 (8·8%) [13]
Fatigue	1 (0·8%) [1]	1 (0·8%) [2]				2 (1·6%) [3]
Chest pain	2 (1·6%) [2]					2 (1·6%) [2]

Chills	1 (0·8%) [1]					1 (0·8%) [1]
Facial pain	1 (0·8%) [1]					1 (0·8%) [1]
Feeling hot	1 (0·8%) [1]					1 (0·8%) [1]
**Metabolism and nutrition disorders**	**20 (16·0%) [21]**	**4 (3·2%) [5]**		**1 (0·8%) [1]**		**24 (19·2%) [27]**
Decreased appetite	20 (16·0%) [20]	4 (3·2%) [4]				24 (19·2%) [24]
Hyperkalaemia		1 (0·8%) [1]		1 (0·8%) [1]		1 (0·8%) [2]
Dehydration	1 (0·8%) [1]					1 (0·8%) [1]
**Psychiatric disorders**	**15 (12·0%) [19]**	**4 (3·2%) [6]**	**2 (1·6%) [2]**			**19 (15·2%) [27]**
Insomnia	9 (7·2%) [11]	3 (2·4%) [3]				12 (9·6%) [14]
Psychotic disorder	1 (0·8%) [1]		2 (1·6%) [2]			3 (2·4%) [3]
Agitation	2 (1·6%) [2]					2 (1·6%) [2]
Anxiety	1 (0·8%) [1]	1 (0·8%) [1]				2 (1·6%) [2]
Hallucination	2 (1·6%) [2]					2 (1·6%) [2]
Logorrhoea	1 (0·8%) [1]	1 (0·8%) [1]				2 (1·6%) [2]
Abnormal behaviour		1 (0·8%) [1]				1 (0·8%) [1]
Crying	1 (0·8%) [1]					1 (0·8%) [1]
**Investigations**	**3 (2·4%) [3]**	**14 (11·2%) [16]**	**4 (3·2%) [4]**	**1 (0·8%) [2]**		**20 (16·0%) [25]**
Blood potassium increased		5 (4·0%) [5]	3 (2·4%) [3]			8 (6·4%) [8]
Blood potassium decreased		3 (2·4%) [3]	1 (0·8%) [1]	1 (0·8%) [1]		5 (4·0%) [5]
Blood calcium decreased		2 (1·6%) [2]		1 (0·8%) [1]		3 (2·4%) [3]
Romberg test positive	3 (2·4%) [3]					3 (2·4%) [3]
Blood albumin decreased	1 (0·8%) [1]					1 (0·8%) [1]
Blood bilirubin increased		1 (0·8%) [1]				1 (0·8%) [1]
Blood glucose increased		1 (0·8%) [1]				1 (0·8%) [1]
Blood phosphorus increased		1 (0·8%) [1]				1 (0·8%) [1]
Blood sodium decreased		1 (0·8%) [1]				1 (0·8%) [1]
Blood sodium increased		1 (0·8%) [1]				1 (0·8%) [1]
**Blood and lymphatic system disorders**	**4 (3·2%) [4]**	**10 (8·0%) [11]**	**7 (5·6%) [7]**			**20 (16·0%) [22]**
Anaemia	1 (0·8%) [1]	8 (6·4%) [9]	6 (4·8%) [6]			14 (11·2%) [16]
Neutropenia	2 (1·6%) [2]	2 (1·6%) [2]	1 (0·8%) [1]			5 (4·0%) [5]
Splenomegaly	1 (0·8%) [1]					1 (0·8%) [1]
**Infections and infestations**	**4 (3·2%) [4]**	**4 (3·2%) [5]**	**7 (5·6%) [7]**			**13 (10·4%) [16]**
Malaria	2 (1·6%) [2]	3 (2·4%) [3]	6 (4·8%) [6]			10 (8·0%) [11]
Fungal infection	1 (0·8%) [1]	1 (0·8%) [1]				2 (1·6%) [2]
Cerebral malaria			1 (0·8%) [1]			1 (0·8%) [1]
Nasopharyngitis	1 (0·8%) [1]					1 (0·8%) [1]
Typhoid fever		1 (0·8%) [1]				1 (0·8%) [1]

**Musculoskeletal and connective tissue disorders**	**10 (8·0%) [11]**	**4 (3·2%) [4]**				**13 (10·4%) [15]**
Back pain	7 (5·6%) [8]	4 (3·2%) [4]				10 (8·0%) [12]
Neck pain	3 (2·4%) [3]					3 (2·4%) [3]
**Eye disorders**	9 (7·2%) [9]	1 (0·8%) [1]				10 (8·0%) [10]
Conjunctivitis	3 (2·4%) [3]					3 (2·4%) [3]
Eye pain	1 (0·8%) [1]	1 (0·8%) [1]				2 (1·6%) [2]
Eyelid oedema	2 (1·6%) [2]					2 (1·6%) [2]
Photophobia	2 (1·6%) [2]					2 (1·6%) [2]
Eye pruritus	1 (0·8%) [1]					1 (0·8%) [1]
**Respiratory, thoracic and mediastinal disorders**	**4 (3·2%) [5]**	**2 (1·6%) [2]**			**1 (0·8%) [1]**	**6 (4·8%) [8]**
Cough	2 (1·6%) [2]	1 (0·8%) [1]				3 (2·4%) [3]
Dyspnoea	1 (0·8%) [2]				1 (0·8%) [1]	1 (0·8%) [3]
Productive cough	1 (0·8%) [1]					1 (0·8%) [1]
Rhinorrhoea		1 (0·8%) [1]				1 (0·8%) [1]
**Skin and subcutaneous tissue disorders**	**7 (5·6%) [8]**					**7 (5·6%) [8]**
Pruritus	5 (4·0%) [5]					5 (4·0%) [5]
Hyperhidrosis	1 (0·8%) [1]					1 (0·8%) [1]
Pruritus generalised	1 (0·8%) [1]					1 (0·8%) [1]
Rash papular	1 (0·8%) [1]					1 (0·8%) [1]
**Cardiac disorders**	5 (4·0%) [6]					5 (4·0%) [6]
Palpitations	3 (2·4%) [4]					3 (2·4%) [4]
Arrhythmia	2 (1·6%) [2]					2 (1·6%) [2]
**Immune system disorders**	**1 (0·8%) [1]**	**1 (0·8%) [1]**				**1 (0·8%) [2]**
Hypersensitivity	1 (0·8%) [1]	1 (0·8%) [1]				1 (0·8%) [2]
**Injury, poisoning and procedural complications**	**1 (0·8%) [1]**				**1 (0·8%) [1]**	**2 (1·6%) [2]**
Injury					1 (0·8%) [1]	1 (0·8%) [1]
Procedural pain	1 (0·8%) [1]					1 (0·8%) [1]
**Vascular disorders**	**1 (0·8%) [1]**					**1 (0·8%) [1]**
Hot flush	1 (0·8%) [1]					1 (0·8%) [1]

Data are presented as the number of patients (percent of patients) [number of events]. Dictionary used MedDRA version 16·1. ITT, intention-to-treat; MedDRA, Medical Dictionary for Regulatory Activities. CTCAE grade 1, 2, 3, 4 and 5 corresponding to mild, moderate, severe, life-threatening and death.

Protocol summary S6

**Study Title**	Efficacy and Safety of Fexinidazole in Children at Least 6 Years of Age and Weighing Over 20 kg with Human African Trypanosomiasis (HAT): a prospective, multicentre, open-label study, plug-in to the pivotal study
**Study Phase**	II/III
**Indication**	Human African Trypanosomiasis (HAT) due to *Trypanosoma brucei gambiense* (all stages)
**Protocol number**	DNDiHATFEX006
**Study Rationale**	HAT is a potentially fatal, neglected disease.<br>HAT is caused by a parasite that initially invades the blood, the lymph nodes and then the central nervous system. At the latter stage, the treatment of HAT requires adequate drug concentrations in the brain.<br><br>The clinical presentation of HAT is identical in children and adults, and the reference treatments are also the same as those used in adults, i.e. pentamidine for patients at stage 1 and the association nifurtimox-eflornithine (NECT) for patients at early and late stage 2.<br><br>The present will be a plug-in to the pivotal study DNDiFEX004 in adults, which means that it will be possible to compare the results from the two studies since patients will be recruited simultaneously i.e. same centres and investigators, genetically homogeneous population.<br><br>As of 21 November 2013, 188 patients, i.e. around 125 patients receiving fexinidazole, had been randomised in the DNDiFEX004 study. No safety issues were identified on blinded data review. A total of 11 serious adverse events were reported, 3 of which were considered as possibly related to the investigational product and which resolved without sequelae. Overall, the safety profile was similar to that observed in earlier studies in healthy volunteers, and there were no treatment discontinuations. A meeting of the Data and Safety Monitoring Board was held. No safety issues were identified, and it was recommended to continue the study with the same design. No parasites were found in any patients at the End of Treatment visit, based on blinded review, and no relapses were observed among 80 patients who attended their 6-month follow-up visit, with the exception of one patient whose health status had been poor at inclusion and who died shortly after leaving hospital (blind not lifted).<br><br>In the DNDiFEX004 study, fexinidazole is administered by the oral route once daily after a meal: three 600-mg tablets daily for 4 days, followed by two 600-mg tablets daily for the next 6 days.<br><br>Pharmacokinetic analysis of the first 39 patients treated with fexinidazole in the DNDiFEX004 study showed that mean exposure to the M2 metabolite, the more active metabolite, in the cerebrospinal fluid was 2.6 times higher than the minimum inhibitory concentration used as the target value based on animal data collected in preclinical efficacy studies. Blood M1 and M2 concentrations were higher in patients than in healthy volunteers. The cerebrospinal fluid-to-blood ratio was around 31% for M2 and 52% for M1.

	In order to provide the results within a reasonable timeframe, the study will be open label and will include a total of 125 children 6 years of age or older with a body weight of at least 20 kg. The patients will undergo the same assessments and investigations as the adult patients in Study DNDiFEX004. Under these conditions, if there are no statistically significant differences with regard to the efficacy and safety data, the results of treatment in children will be considered to be equivalent to those in adults. Thus, the number of children needed to treat is lower than if this had been a stand-alone study.<br><br>The objective is to be able to administer fexinidazole as a single treatment to all patients with HAT, without recourse to CSF-based staging of the disease. The aim of the present study is to assess the efficacy and safety of fexinidazole in children with stage-1, early stage-2 and late stage-2 HAT, stratified into three sub-groups. The study will provide an assessment of the success rate of treatment overall and by stratum, as well allowing for comparison with historical data on pentamidine in patients with stage-1 HAT.
**Choice of dose**	The dosing regimen is as follows:<br>• Body weight ≥ 20 kg and < 35 kg:<br>o 1200 mg from Day 1 to Day 4<br>o 600 mg from Day 5 to Day 10<br>• Body weight ≥ 35 kg (same regimen as for adults)<br>o 1800 mg from Day 1 to Day 4<br>o 1200 mg from Day 5 to Day 10
**Study Objectives**	The objective of the study is to assess the efficacy and safety of an oral dosing regimen involving one daily intake for 10 days in the treatment of HAT due to *T.b. gambiense* at stage 1 or 2 in children 6 years of age or older weighing more than 20 kg.<br><br>**Primary Objective**<br>▪ To demonstrate that the success rate 12 months after the end of treatment in patients with stage-1 or stage-2 HAT is greater than an acceptable rate of 80% and consistent with a target rate of 92%.<br><br>**Secondary Objectives**<br>▪ To verify whether the success rate varies depending on the stage of the disease; if the success rate is significantly different between the 3 strata, to show that the rate is greater than 80% and consistent with the historical success rate reported with NECT in patients with stage-2 HAT and with pentamidine in patients with stage-1 HAT.<br>▪ To verify whether the success rate of treatment with fexinidazole varies depending on the WBC count in the CSF prior to treatment initiation.<br>▪ To study changes in the success rate over time.
**Primary Endpoint**	**Efficacy**<br><br>The primary efficacy endpoint is the outcome, i.e. success or failure, observed at the test-of-cure visit, 12 months after the end of treatment.

	Success means that the patient is cured, as defined by adapted WHO criteria.<br><br>In addition, although the timepoint for assessment of the efficacy endpoint was set at 12 months after the end of treatment, patients will be followed until at least 18 months after the end of treatment.
**Secondary Endpoints**	**<u>Efficacy</u>**<br><br>Outcome, i.e. success or failure, at each visit between the end of treatment and 18 months.
	**<u>Safety</u>**<br><br>• Occurrence of any adverse events grade ≥ 3, including laboratory abnormalities, during the observation period. Adverse events will be graded according to the Common Toxicity Criteria for Adverse Events of the National Cancer Institute, version 4.03.<br>• Occurrence of any adverse events, at any grade, during the observation period.<br>• Occurrence of any treatment-related adverse events (grade ≥ 3 and any grade) during the observation period.<br>• Occurrence of any serious adverse events between the first intake of the IP and the end of the follow-up period (18 months).
	**<u>Electrocardiogram (ECG) Endpoints</u>**<br>Categories of QT/QTc and changes on ECG tracings recorded at various timepoints.
	**<u>Pharmacokinetic (PK) Endpoints</u>**<br><br>A series of blood samples for PK testing will be collected using dry blood spot, as well as a dry cerebrospinal fluid spot.<br><br>In order to adjust to the patients' ages, the number of blood samples has been reduced, i.e. 2 samples less than adults, as follows:<br><br>Whole blood:<br>• On Day 10: 3 hours and 7 hours 15 minutes after the last intake of fexinidazole<br>• On Day 11: 24 hours after the last intake of fexinidazole<br>• On Day 12: 48 h hours after the last intake of fexinidazole.<br>Cerebrospinal fluid:<br>• On Day 11: 24 hours after the last intake of fexinidazole<br>The lumbar puncture at D11, and therefore the PK analysis on cerebrospinal fluid was stopped in October 2014 after approximately 30 patients, as initially planned in the protocol.

**Study Design**	This is an open-label, single-group, multicentre, Phase II/III study. The study will be a plug-in to the pivotal study, DNDiFEX004, and will be performed at the same sites.
**Inclusion and Exclusion Criteria**	**Inclusion criteria:**<br><br>▪ Signed informed consent form from one parent or from the legal representative<br>▪ Assent from the child to participate in the study, collected in the presence of an impartial witness<br>▪ Between 6 and 15 years of age<br>▪ Body weight at least 20 Kg<br>▪ Male or female<br>▪ Able to ingest at least one complete meal per day (or at least one sachet of Plumpy'Nut®)<br>▪ Able to swallow the 600-mg tablets of fexinidazole<br>▪ Karnofsky score > 50<br>▪ Evidence of trypanosomes in blood and/or lymph and/or cerebrospinal fluid.<br>▪ Having a permanent address and able to comply with the schedule of follow-up visits.<br><br>**Exclusion Criteria:**<br><br>▪ Refusal to participate in the study, expressed by child<br>▪ Body weight strictly less than 20 Kg;<br>▪ Severe malnutrition, defined as Body Mass Index < 16. (–2 standard deviation);<br>▪ Unable to take medication by the oral route;<br>▪ Pregnancy or breast-feeding ;<br>▪ Clinically significant medical condition (other than HAT) that could, in the opinion of the Investigator, jeopardise the patient's safety or interfere with participation in the study, including, but not limited to significant liver or cardiovascular disease, suspected or proven active infection (including HIV infection), CNS trauma or seizure disorder, coma or consciousness disturbances;<br>▪ Severely deteriorated general status, including as a result of cardiovascular shock, respiratory distress or end-stage disease;<br>▪ Any condition (excluding HAT-specific symptoms) that affects the patient's ability to communicate with the Investigator as required to complete the study;

	▪ Any contraindication to imidazole drugs, i.e. known hypersensitivity to imidazoles;<br>▪ Prior treatment for HAT in the previous 2 years;<br>▪ Prior enrolment in the study or prior intake of fexinidazole;<br>▪ Foreseeable difficulty complying with follow-up, including family of migrant workers, refugee status, itinerant trader, etc.;<br>▪ Active alcohol or drug addiction.<br>▪ Clinically significant laboratory test abnormality, with:<br>o alanine aminotransferase and/or aspartate aminotransferase more than 2 times the upper limit of normal (ULN),<br>o total bilirubin more than 1.5 x ULN,<br>o severe leukopenia at < $2000/mm^3$,<br>o potassium < 3.5 mmol/L,<br>o any other clinically significant laboratory test abnormality (see Investigator manual for details);<br>▪ Pregnancy confirmed by a positive urine pregnancy test within 24 hours prior to the start of treatment (see Section 5.8.3 Contraception) – for girls ≥ 12 years of age;<br>▪ ECG abnormality as assessed by central cardiologist;<br>▪ QTcF interval ≥ 450 msec on automatic reading, if the first reading is abnormal, a second reading will be performed at least 10 to 20 min after placing the patient in the resting position;<br>▪ Not tested for malaria and/or not having received appropriate treatment for malaria<br>▪ Not having received appropriate treatment for soil-transmitted helminthiasis
**Study Duration**	Each patient's participation will last approximately 19 months and will include:<br>• Pre-treatment period<br>• Treatment period of 10 days<br>• Hospitalisation for 3 to 7 days after treatment<br>• Additional follow-up visit at 9 weeks after D1 (between D64 and D70)<br>• Out-patient follow-up with visits at 3, 6, 12, and 18 months.

**Investigational Product**	**<u>Investigational Product</u>**<br>Fexinidazole, 600-mg tablets to be taken by the oral route after the main meal:<br>• Patients with body weight ≥ 20 kg and < 35 kg:<br>o 1200 mg (2 tablets) in one daily intake for 4 days, followed by<br>o 600 mg (1 tablet) in one daily intake for the next 6 days.<br>• Patients with body weight ≥ 35 kg:<br>o 1800 mg (3 tablets) in one daily intake for 4 days, followed by<br>o 1200 mg (2 tablets) in one daily intake for the next 6 days.<br>The total duration of treatment will be 10 days.
**Statistical Analyses**	**<u>Analysis Sets</u>**<br>Analysis of the primary efficacy endpoint will be performed on the intention-to-treat population.<br>• Intention-to-treat population: all patients who received at least one dose of fexinidazole.<br>• Per-protocol population: all patients with no major protocol violations that could interfere with the efficacy analysis.<br>Sensitivity analyses will be performed on treatment completers, the population of evaluable patients and the per-protocol population.<br>**<u>Analysis</u>**<br>The primary analysis will be performed on the ITT population, by estimating the rate of success or failure at 12 months of follow-up. If the lower bound of the 95% confidence interval is less than or equal to 80% the study will be a failure, otherwise it will be a success. The primary test is an exact test derived from the exact confidence interval of the success rate at 12 months.<br>**<u>Sample Size</u>**<br>Determination of the sample size was based on the primary analysis. The sample size must be 125 patients having received at least one dose of fexinidazole. The sample size may be 126 patients if two patients are recruited simultaneously at the time of study discontinuation, however, it must not exceed 126 patients.
